# Interferon-γ and CXCL10 responses related to complaints in patients with Q fever fatigue syndrome

**DOI:** 10.1007/s10096-018-3265-z

**Published:** 2018-05-26

**Authors:** Ruud P. H. Raijmakers, Anne F. M. Jansen, Stephan P. Keijmel, Teske Schoffelen, Anja Scholzen, Jos W. M. van der Meer, Leo A. B. Joosten, Mihai G. Netea, Marcel van Deuren, Chantal P. Bleeker-Rovers

**Affiliations:** 1 Radboud Expertise Center for Q fever, Department of Internal Medicine, Division of Infectious Diseases 463, Radboud University Medical Center, P.O. Box 9101, 6500 HB Nijmegen, The Netherlands; 2 Department of Internal Medicine, Radboud University Medical Center, P.O. Box 9101, 6500 HB Nijmegen, The Netherlands; 3 Innatoss, 5349 AB Oss, The Netherlands; 4 Radboud Center for Infectious Diseases, Radboud University Medical Center, P.O. Box 9101, 6500 HB Nijmegen, The Netherlands

**Keywords:** Cell-mediated immunity, *Coxiella burnetii*, Interferon-gamma, CXCL10, Q fever, Q fever fatigue syndrome

## Abstract

Approximately 20% of patients with acute Q fever develop Q fever fatigue syndrome (QFS), a debilitating fatigue syndrome. This study further investigates the role of *C. burnetii*-specific IFNγ, but also IL-2, CXCL9, CXCL10, and CXLC11 production in QFS patients. *C. burnetii*-specific IFNy, IL-2, CXCL9, CXCL10, and CXCL11 production were tested in ex vivo stimulated whole blood of QFS patients who recovered from their complaints (*n* = 8), QFS patients with persisting complaints (*n* = 27), and asymptomatic Q fever seropositive controls (*n* = 10). With the exclusion of one outlier, stimulation with *C. burnetii* revealed significantly higher IFNy and CXCL10 production in QFS patients with persisting complaints (medians 288.0 and 176.0 pg/mL, respectively) than in QFS patients who recovered from their complaints (medians 93.0 and 85.5 pg/mL, respectively) (*p* = 0.041 and 0.045, respectively). No significant differences between groups were found for *C. burnetii*-specific IL-2, CXCL9, and CXCL11 production. These findings point towards a difference in cell-mediated immunity in QFS patients with persisting complaints compared to those who recovered from their complaints. Such a difference may aid to eventually diagnose QFS more objectively and might serve as an indicator of its underlying etiology.

## Introduction

Q fever is a zoonotic disease caused by the Gram-negative intracellular bacterium *Coxiella burnetii* that occurs worldwide [1]. Human primary infection is thought to remain asymptomatic in approximately 60% of cases with the other 40% resulting in symptomatic infection. Symptomatic primary infection with *C. burnetii* is called acute Q fever and usually presents as a self-limiting flu-like illness, pneumonia or hepatitis [1]. Approximately 20% of acute Q fever patients develop a prolonged state of debilitating post-infectious fatigue, called Q fever fatigue syndrome (QFS) [2–4]. Contrary to chronic Q fever, a persistent and life-threatening infection of pre-existing vascular and/or valvular lesions or prostheses [5], no signs of active infection are found in QFS. During the Q fever outbreak in the Netherlands between 2007 and 2011, over 4000 acute Q fever cases were notified, and it is estimated that at least 32,200 people were infected [6]. Ever since, hundreds of patients have been diagnosed with QFS [6, 7].

Diagnosing QFS is challenging and requires careful clinical evaluation and exclusion of chronic Q fever and otherwise causative somatic and psychiatric diseases [Dutch guideline on Q fever fatigue syndrome, National Institute for Public Health and the Environment]. Testing for *C. burnetii*-specific antibodies has an important role in diagnosing QFS. Not only for excluding chronic Q fever but also to confirm a past Q fever infection. Unfortunately, *C. burnetii*-specific antibody titers are not related to perceived complaints during QFS and are therefore unable to differentiate QFS from a past Q fever infection. A recent study by our group, however, showed altered cell-mediated immunity in QFS patients that might help diagnose them in a more objective manner [8]. The *C. burnetii*-specific whole-blood interferon-γ (IFNγ) production assay showed promising results in differentiating QFS patients from Q fever seropositive controls and further implementation of an IFNγ/interleukin-2 (IL-2) ratio helped differentiate QFS patients from chronic Q fever patients [8]. Furthermore, a recent study has shown a promising role for chemokines CXCL9, CXCL10, and CXCL11 as potential new biomarkers for persistent infection with *C. burnetii*, i.e. chronic Q fever [9].

This study further investigates the role of the *C. burnetii*-specific whole-blood IFNγ and IL-2 production assay and adds CXCL9, CXCL10, and CXCL11, as possible biomarkers for perceived complaints in QFS. We start by comparing antigen-specific whole-blood IFNγ and IL-2 production in QFS patients who have recovered from their complaints, QFS patients who have persisting complaints, and asymptomatic Q fever seropositive controls. Finally, by adding antigen-specific whole-blood CXCL9, CXCL10, and CXCL11 production to this assay, we examine whether we can enhance biologic differentiation between these groups.

## Materials and methods

### Study population

The study population consisted of QFS patients (*n* = 35) and a group of asymptomatic Q fever seropositive controls (*n* = 10). All QFS patients were diagnosed at the Radboud Expertise Center for Q fever, Nijmegen, the Netherlands, after a uniform work-up according to the Dutch guideline on QFS. At diagnosis, all QFS patients met the following criteria: (i) fatigue lasting ≥ 6 months; (ii) sudden onset of severe fatigue, defined as a score ≥ 35 on the subscale fatigue severity of the Checklist Individual Strength (CIS), or significant increase in fatigue related to a symptomatic acute Q fever infection; (iii) chronic Q fever and other causes of fatigue, somatic or psychiatric, were excluded; and (iv) fatigue resulted in significant functional impairment, defined as a total score ≥ 450 on the Sickness Impact Profile (SIP). All patients who were recruited for this study primarily participated in the Qure study [10]. In the Qure study, 154 men and non-lactating women with QFS aged ≥ 18 years were equally randomized with a 1:2 ratio between two treatment groups: cognitive behavioral therapy (CBT) (*n* = 50) and medication (*n* = 104). In the medication group, a second double-blinded randomization was performed between doxycycline and placebo treatment [10]. One year after completion of the Qure study, patients who were allocated to the CBT and placebo treatment groups were asked to donate blood (5 mL) at a local health center and fill-out CIS questionnaires for this study. A cut-off score of 35 on the CIS subscale Fatigue Severity was used to differentiate recovered QFS patients (CIS subscale Fatigue Severity score < 35) from QFS patients with persistent complaints (CIS subscale Fatigue Severity score ≥ 35). Asymptomatic Q fever seropositive controls who previously participated in the ‘Q-Herpen-II’ study were contacted by mail and asked to donate blood (5 mL) at a local health center [11].

### In vitro whole blood stimulation

The cellular immune response to *C. burnetii* was measured using a commercially available whole blood Q fever IFNγ release assay, Q-detect (Innatoss, Oss, the Netherlands), according to the instructions of the manufacturer.

Q-detect utilizes a heat-killed *C. burnetii* antigen prepared at Wageningen Bioveterinary Research (WBR) in Lelystad (the Netherlands). The antigen is based on a proprietary strain, *C. burnetii* 2009-02629, which was isolated at the WBR from a goat placenta during the Dutch Q fever outbreak in 2009. The strain was cultured in axenic medium. Culture methods were based on Omsland et al. [12]; bacteria were collected by centrifugation, washed in PBS, and heat-killed for 30 min at 99 °C. The antigen preparation was calibrated against a similar preparation of the reference Nine Mile strain [13].

For Q-detect, whole blood is collected in lithium heparin-coated tubes (Vacutainer, Becton Dickinson). Within 12 h after blood collection, 180 μL whole blood is mixed with 20 μL heat-killed *C. burnetii* in a polypropylene 96-well plate (Greiner). As respectively negative and positive controls, RPMI medium (ThermoScientific) and phytohemagglutinin (ThermoScientific, 1.5% final concentration) were used. Plates were sealed and incubated for 22–24 h at 37 °C, after which plates were kept at 2–8 °C. After incubation, supernatants were collected and stored at − 20 °C until cytokine measurement.

### Cytokine measurements

IFNγ production was measured by enzyme-linked immunosorbent assay (ELISA; Pelikine compact, Sanquin, Amsterdam, the Netherlands), according to the manufacturer’s instructions. IL-2, CXCL9, CXCL10, and CXCL11 were measured using a multiplex beads assay (Bio-Rad, CA, USA) according to the manufacturer’s instructions.

### Ethical statement

All participants provided written informed consent and the study was approved by the Medical Ethical Review Committee of the Arnhem-Nijmegen region.

### Statistical analysis

Data were analyzed using Graphpad Prism (Graphpad Software Inc., version 5.03) and SPSS (Version 22.0, SPSS, Inc). Either the Mann-Whitney *U* test or Kruskal-Wallis test were used as non-parametric tests to determine differences between groups. The correlation between patient characteristics and IFNγ, IL-2, CXCL9, CXCL10, and CXCL11 production was determined with the non-parametric Spearman’s rank correlation coefficient. Statistical significance was attained if *p* < 0.05.

## Results

### Patients and controls

At the time of blood collection, QFS patients had either recovered from their complaints (*n* = 8) or had persisting complaints (*n* = 27), depending on their score on the CIS subscale Fatigue Severity. Asymptomatic Q fever seropositive controls (*n* = 10) had IgG phase I or II titers of ≥ 1:64 in 2014 but did not fulfill the Dutch Consensus Guidelines for chronic Q fever [14]. At the time of blood collection, all asymptomatic Q fever seropositive controls scored < 35 on the CIS subscale Fatigue Severity. The mean age of QFS patients with persisting complaints was 50.7 years (standard deviation (SD) 11.9), which did not significantly differ from 52.8 (SD 12.8) and 53.4 (SD 10.6) for QFS patients who recovered from their complaints and asymptomatic Q fever seropositive controls (*p* = 0.794), respectively. Gender distribution also did not differ significantly between groups (*p* = 0.841) (Table 1).

**Table 1:** Baseline characteristics of patients with Q fever fatigue syndrome (QFS) and asymptomatic Q fever seropositive controls

	QFS-recovered (*n* = 8)	QFS-persisting complaints (*n* = 27)	Q fever seropositive controls (*n* = 10)
Female gender (%)	5 (62.5)	16 (59.3)	5 (50.0)
Mean age, years (±SD)	52.8 (± 12.8)	50.7 (11.9)	53.4 (10.6)
Mean CIS subscale Fatigue Severity (±SD)	22.0 (14.3–29.0)	49.0 (43.0–53.0)	17.5 (15.0–35.0)
Median symptom duration, months (IQR)^a^	29.5 (23.8–52.0)	92.0 (84.0–104.0)	–
Placebo (%)^b^	4 (50.0)^c^	14 (51.9)^d^	–
CBT (%)^b^	4 (50.0)	13 (48.1)	–

*QFS* Q fever fatigue syndrome, *SD* standard deviation, *CIS* Checklist Individual Strength, *IQR* interquartile range, *CBT* cognitive behavioral therapy

^a^Symptom duration: time onset of symptoms until blood sampling

^b^Allocated treatment during the Qure study

^c^One out of four followed additional CBT treatment

^d^Two out of 14 followed additional CBT treatment

### IFNγ and IL-2 production

**Fig. 1:**
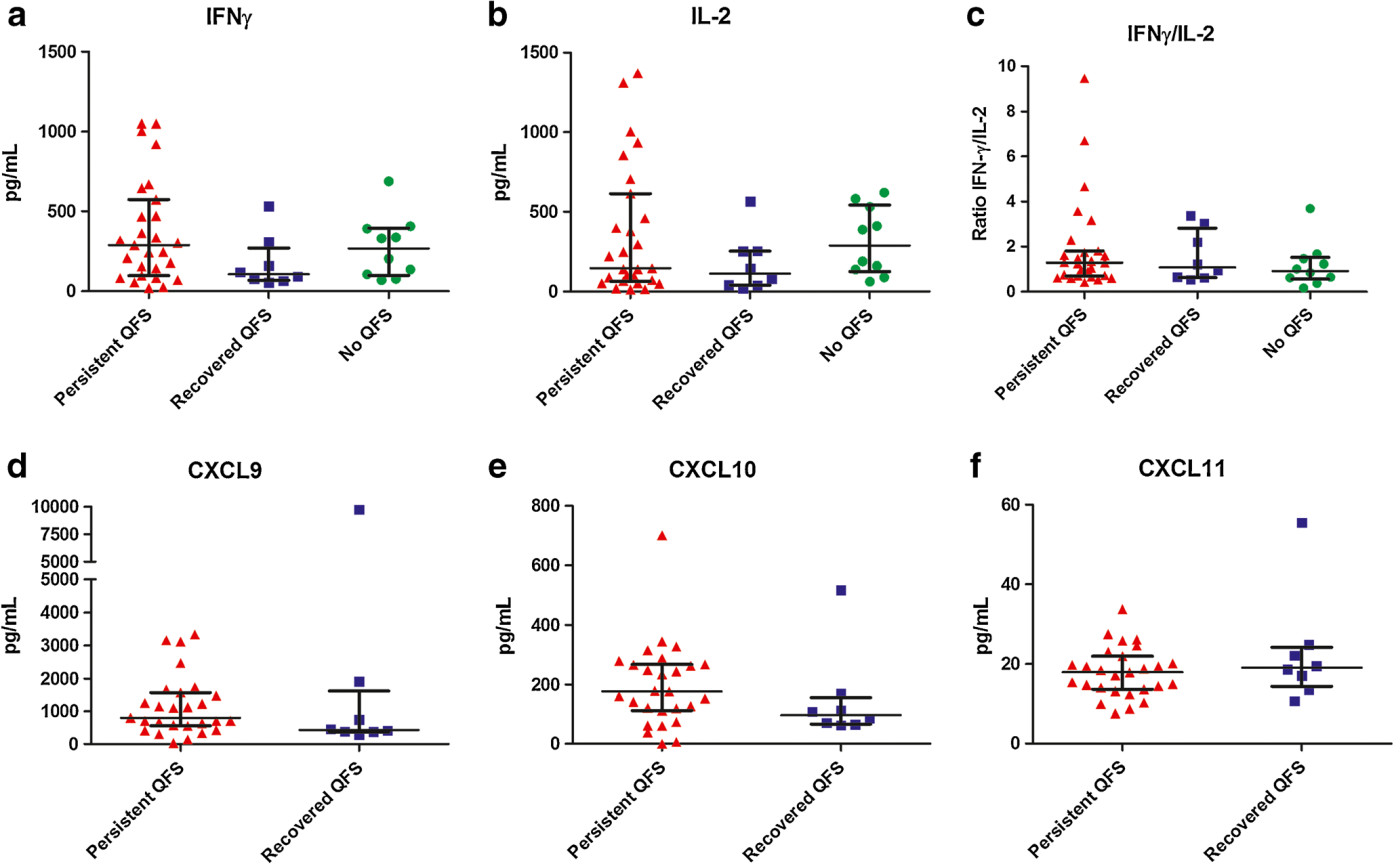
IFNγ, IL-2, CXCL9, CXCL10, and CXCL11 production in Q fever fatigue syndrome (QFS) patients and asymptomatic Q fever seropositive controls. **a** *C.b.* 2009-02629-induced net IFNγ production after 24 h incubation of whole blood, showing no significant difference in IFNγ production between QFS patients with persisting complaints, QFS patients who recovered from their complaints, and asymptomatic Q fever seropositive controls. **b** *C.b.* 2009-02629-induced net IL-2 production after 24 h incubation of whole blood, showing no significant difference in IL-2 production between QFS patients with persisting complaints, QFS patients who recovered from their complaints, and asymptomatic Q fever seropositive controls. **c** IFNγ/IL-2 ratio, showing no significant difference between QFS patients with persisting complaints, QFS patients who recovered from their complaints, and asymptomatic Q fever seropositive controls. **d** *C.b.* 2009-02629-induced net CXCL9 production after 24 h incubation of whole blood, showing no significant difference in CXCL9 production between QFS patients with persisting complaints and QFS patients who recovered from their complaints. **e** *C.b.* 2009-02629-induced net CXCL10 production after 24 h incubation of whole blood, showing no significant difference in CXCL10 production between QFS patients with persisting complaints and QFS patients who recovered from their complaints. **f** *C.b.* 2009-02629-induced net CXCL11 production after 24 h incubation of whole blood, showing no significant difference in CXCL11 production between QFS patients with persisting complaints and QFS patients who recovered from their complaints. Median ± IQR are shown. The Mann-Whitney and Kruskall-Wallis test were used. Figure was made in Gaphpad Prism. Abbreviations: *IFNγ* = interferon-gamma; *IL* = interleukin; *CXCL* = C-X-C ligand; *QFS* = Q fever fatigue syndrome; *C.b.* = *Coxiella burnetii*; *Recovered QFS* = QFS patients who recovered from their complaints; *Persistent QFS* = QFS patients with persisting complaints; *No QFS* = asymptomatic Q fever seropositive controls; *IQR* = interquartile range

Stimulation with *C. burnetii* for 24 h yielded a median IFNγ production of 288 pg/mL for QFS patients with persisting complaints, 107 pg/mL for QFS patients who recovered from their complaints, and 267 pg/mL for asymptomatic Q fever seropositive controls, but showed no significant difference between groups. No significant difference was observed in median IL-2 production between QFS patients with persisting complaints (median 147 pg/mL), QFS patients who recovered from their complaints (median 114 pg/mL), and asymptomatic Q fever seropositive controls (median 289 pg/mL). Converting IFNγ and IL-2 production to an IFNγ/IL-2 ratio for each subject also did not significantly differentiate between groups (Table 2 and Fig. 1a–c).

**Table 2:** Median *C. burnetii*-specific production of IFNγ and IL-2 in patients with Q fever fatigue syndrome (QFS) and asymptomatic Q fever seropositive controls, together with production of CXCL9, CXCL10, and CXCL11 in QFS patients

	QFS-recovered (*n* = 8)	QFS-persistent complaints (*n* = 27)	Q fever seropositive controls (*n* = 10)
IFNγ production, pg/mL (IQR)	107 (69–271)	288 (98–574)	267 (100–395)
Nil, pg/mL (IQR)	5 (2–7)	5 (3–7)	4 (3–12)
IL-2 production, pg/mL (IQR)	114 (40–253)	147 (66–614)	289 (127–543)
Nil, pg/mL (IQR)	0 (0–1)	0 (0–0)	68 (5–117)
Ratio IFNγ/IL-2 (IQR)	1 (1–3)	1 (1–2)	1 (1–2)
CXCL9 production, pg/mL (IQR)	436 (378)	803 (558–1572)	–
Nil, pg/mL (IQR)	217 (141–718)	171 (131–292)	–
CXCL10 production, pg/mL (IQR)	97 (66–156)	176 (112–267)	–
Nil, pg/mL (IQR)	57 (47–69)	63 (37–102)	–
CXCL11 production, pg/mL (IQR)	19 (14–24)	18 (14–22)	–
Nil, pg/mL (IQR)	12 (10–22)	9 (7–12)	–

Median IFNγ, IL-2, CXCL9, CXCL10, and CXCL11 production and IFNγ/IL-2 ratio after 24-h incubation of whole blood with *C. burnetii* 2009–02629 or RPMI (Nil)

*IFNγ* interferon-gamma, *IL* interleukin, *CXCL* C-X-C ligand, *QFS* Q fever fatigue syndrome, *IQR* interquartile range, *Nil* negative control, i.e., Roswell Park Memorial Institute medium

As can be seen in Fig. 1a, there is one patient with an *C. burnetii*-specific IFNγ production of 533 pg/mL who is clearly an outlier and continues to behave like this in the other measurements (Fig. 1b–f). Retesting these results produced similar concentrations. Clinically, the patient did not differ in any way from the others within the group of recovered QFS patients.

With the exclusion of this patients, a significantly higher IFNy production was found in QFS patients with persistent complaints (median 288 pg/mL) compared to QFS patients who recovered from their complaints (median 93 pg/mL) (*p* = 0.041). No significant difference between groups was found for *C. burnetii*-specific IL-2 production or IFNγ/IL-2 ratio.

### CXCL9, CXCL10, and CXCL 11 production

Stimulation with *C. burnetii* for 24 h yielded no significant difference in median CXCL10 production between QFS patients with persisting complaints and QFS patients who recovered from their complaints (medians 176 and 97 pg/mL, respectively). No significant differences were found comparing CXCL9 and CXCL11 production between QFS patients with persisting complaints (medians 803 and 18 pg/mL, respectively) and QFS patients who recovered from their complaints (medians 436 and 19 pg/mL, respectively) (Table 2 and Fig. 1d–f).

When excluding the same patient as for the analysis above, stimulation with *C. burnetii* for 24 h revealed significantly higher CXCL10 production in QFS patients with persisting complaints (median 176 pg/mL) than in QFS patients who recovered from their complaints (median 86 pg/mL) (*p* = 0.045). No significant difference between groups was found for *C. burnetii*-specific CXCL9 and CXCL11 production.

### Correlations between patient characteristics and cytokine and chemokine measurements

Correlations between several patient characteristics and cytokine and chemokine measurements were investigated. No correlation was found between IFNγ, IL-2, CXCL9, CXCL10, or CXCL11 production and level of fatigue or symptom duration, regardless of the one outlier.

## Discussion

In this study, we investigated the relation of antigen-specific biomarkers IFNγ, IL-2, CXCL9, CXCL10, and CXCL11 to perceived complaints in QFS. Except for one outlier, both antigen-specific IFNγ (*p* = 0.041) and CXCL10 (*p* = 0.045) production were higher in QFS patients with persisting complaints, than in those who recovered from their complaints. No significant difference in antigen-specific IFNγ and IL-2 production, and IFNγ/IL-2 ratio, was found between QFS patients and asymptomatic Q fever seropositive controls, or in antigen-specific IL-2, CXCL9, and CXCL11 production between QFS patients with persisting complaints and QFS patients who recovered from their complaints. These results point towards an altered cell-mediated immunity between these groups and indicate that both antigen-specific IFNγ and CXCL10 production could serve as potential biomarkers for perceived complaints in QFS.

Antigen-specific production of IFNγ and IL-2 mainly originates from sensitized effector and memory T cells [15], whereas CXCL9, CXCL10, and CXCL11 are chemokines, inducible by IFNγ, produced by various cell types, e.g., lymphocytes and macrophages, but also endothelial cells and fibroblasts [16]. Previously, our group showed that antigen-specific IFNγ production was significantly higher in QFS patients (*n* = 28; 319.4 pg/mL) than in Q fever seropositive controls (*n* = 135; 120 pg/mL) [8]. It was also shown that antigen-specific IFNγ production is a useful tool for diagnosing a previous Q fever infection [13], and adding antigen-specific IL-2 production helped discriminate chronic Q fever patients from Q fever seropositive controls and QFS patients [8, 17]. In this study, antigen-specific IFNγ production was not significantly higher in QFS patients than in asymptomatic Q fever seropositive controls. We did show that it may be possible to discriminate QFS patients with persistent complaints from recovered QFS patients, with the latter showing lower antigen-specific IFNγ production than the former, even surpassing asymptomatic Q fever seropositive controls. Even though a non-significant difference is seen, it was expected that antigen-specific IL-2 production and IFNγ/IL-2 ratio would not be able to discriminate between QFS patients with persisting complaints, QFS patients who recovered from their complaints, and asymptomatic Q fever seropositive controls [8].

An interesting difference between the present cohort of asymptomatic Q fever seropositive controls and the cohort that was published by Keijmel et al. [8] is that the present cohort reported a symptomatic acute Q fever infection during the outbreak, while the group of Keijmel et al. was found seropositive by screening and hence contained a large number of individuals who did not experience a symptomatic acute Q fever infection. Keeping in mind that high IFNγ, and low IL-10, production phenotypes have been associated with a more severe and long-lasting acute illness, it is conceivable that the individuals of the cohort of Keijmel et al. have a lower IFNγ response in the long term, compared to those who experienced a symptomatic acute Q fever infection [18]. It should also be noted that the present cohort of asymptomatic Q fever seropositive controls was recruited from Herpen, a small town in the Netherlands where the outbreak hit hardest. Therefore, although IFNγ responses appear similar between patients with persistent complaints and asymptomatic Q fever seropositive controls in our study, there are several factors that are likely to influence this observation. In order to better understand the kinetics of antigen-specific IFNγ production in QFS patients and asymptomatic Q fever seropositive controls, we suggest measuring IFNγ production longitudinally, starting at the time of infection. Unfortunately, given the current low incidence rates of acute Q fever infections [RIVM–Q fever], such a study is hard to conduct in the Netherlands. As we did not measure longitudinally, we do not know whether the slope of immune reactivity differs between patients who become asymptomatic following their symptomatic acute Q fever infection and those who protract persistent complaints. It is of interest though that we do see somewhat higher IL-2 responses and a lower IFNγ/IL-2 ratio in the asymptomatic Q fever seropositive control group of our study. This is an argument indeed that patients with persistent complaints might differ from those who recover from their acute Q fever infection.

A recent study showed that antigen-specific production of CXCL9 and CXCL11, but not CXCL10, could further help differentiate chronic Q fever patients from Q fever seropositive controls [9]. Our results show that QFS patients who recovered from their complaints have less antigen-specific CXCL10 production than those with persistent complaints. This was to a lesser extent also the case for CXCL9, but not for CXCL11. Unfortunately, we were unable to test antigen-specific CXCL9, CXCL10, and CXCL11 production in asymptomatic Q fever seropositive controls. Nonetheless, as is the case for IFNγ, these results do point towards an altered cell-mediated immunity in between QFS patients with persisting complaints and those who recovered from their complaints and show potential for antigen-specific CXCL10 production to serve as a biomarker for perceived complaints in QFS.

An interesting question that remains is whether recovering from QFS results in a tempered antigen-specific immune response, or complaints persist because of a perpetuating immunologic etiology. The pathophysiology of QFS remains unclear and theories range from chronic immune stimulation to perpetuating compensation-driven and psychogenic factors [19]. It has long been postulated that the etiology of chronic fatigue syndromes might very well be multi-factorial and could differ per patient. If this is the case for QFS, antigen-specific IFNγ and CXCL10 production might help clinicians differentiate in underlying etiology and subsequently better specify therapeutic decision making, i.e., shifting focus towards a more somatic or psychological perpetuation of complaints, for cognitive behavioral therapists [10].

Although this study points towards an altered cell-medicated immunity in QFS patients with persistent complaints compared to those who recovered from their complaints and shows possible roles for antigen-specific IFNγ and CXCL10 to serve as biomarkers for perceived complaints in QFS, there are several limitations. One limitation is the small group size of QFS patients who recovered from their complaints and missing CXCL9, CXCL10, and CXCL11 data for asymptomatic Q fever seropositive controls. For studies like this, in which sensitive biomarkers are tested in difficult-to-define patient populations, it is paramount to have adequate and ample control groups. Unfortunately, we were unable to draft more QFS patients who recovered from their complaints and asymptomatic Q fever seropositive controls. Additionally, due to an interassay coefficient of variability well above 15%, we had to exclude data for antigen-specific CXCL9, CXCL10, and CXCL11 production in asymptomatic Q fever seropositive controls. As the negative control samples of the asymptomatic Q fever seropositive controls were unusually high, we decided this run to be faulty and proceeded with excluding these data from our manuscript. However, even with these limitations, the results of this study give us additional insight in the underlying cell-mediated immunity of perceived complaints in QFS. It also exposes an interesting potential for antigen-specific biomarkers, e.g., IFNγ and CXCL, to help clinicians more objectively diagnose QFS.

## Conclusion

QFS patients with persisting complaints might exhibit a different cell-mediated immunity compared to those who recovered from their complaints. Furthermore, antigen-specific IFNγ and CXCL10 production could serve as potential biomarkers for perceived complaints in QFS and possibly also as indicators of underlying etiology. We recommend further investigation into the role of these biomarkers in the pathophysiology of and diagnostic use for QFS patients with adequately sized control groups.
